# L–carnitine in metabolic dysfunction-associated steatotic liver disease: mechanisms and therapeutic potential

**DOI:** 10.3389/fnut.2026.1804917

**Published:** 2026-03-31

**Authors:** Caili Chen, Jun Zou, Liwei Li, Shikai Liu, Zheng Liu, Li Yao, Peng Peng, Jingrong Liang, Zhijian Chen, Huijian Huang, Jiean Huang

**Affiliations:** 1 Department of Gastroenterology, The Second Affiliated Hospital of Guangxi Medical University, Nanning, Guangxi, China; 2 Department of Nutrition, The Second Affiliated Hospital of Guangxi Medical University, Nanning, Guangxi, China

**Keywords:** insulin resistance, L-carnitine, lipid metabolism, MASLD, mitochondrial function

## Abstract

Metabolic dysfunction-associated steatotic liver disease (MASLD) affects over one-third of the global population, representing one of the most prevalent chronic liver diseases. The escalating global prevalence of MASLD is concerning, as the disease can progress silently to liver fibrosis, cirrhosis, and hepatocellular carcinoma (HCC). Furthermore, MASLD is independently associated with increased risks of cardiovascular disease, chronic kidney disease, and various extrahepatic malignancies, posing a substantial threat to public health. Consequently, developing effective preventive and therapeutic strategies is crucial. L-carnitine is a well-established dietary supplement that demonstrates potential to mitigate the onset and progression of MASLD. Its proposed mechanisms include the enhancement of lipid metabolism, improvement of insulin sensitivity, stabilization of hepatocyte function, and reduction of inflammation, fibrosis, and tumorigenesis. This comprehensive review synthesizes recent advances in the molecular mechanisms through which L-carnitine influences MASLD pathogenesis and critically evaluates its therapeutic potential in MASLD prevention, management, and prognosis.

## 1 Introduction

The global prevalence of non-alcoholic fatty liver disease (NAFLD) has shown a consistent upward trend in recent decades, with current estimates indicating an overall prevalence of 30.05% (1). Significant geographical variations exist. For instance, Latin America exhibits the highest prevalence (44.37%), followed by North Africa and the Middle East (36.53%). In contrast, Western Europe demonstrates the lowest prevalence (25.10%) (1). The insidious disease progression presents substantial clinical challenges, as approximately 20–40% of NAFLD patients may develop non-alcoholic steatohepatitis (NASH) over several years (1, 2), which can subsequently advance to liver fibrosis and HCC (3). NAFLD is independently associated with elevated risks of diabetes (4), cardiovascular disease (5), and chronic kidney disease (6), collectively contributing to a growing global disease burden.

In a pivotal 2023 consensus statement, international liver associations proposed redefining disease terminology, replacing “NAFLD” with “metabolic dysfunction-associated steatotic liver disease (MASLD)” and “NASH” with “metabolic dysfunction-associated steatohepatitis (MASH)” (7). This nomenclature shift aims to better reflect the underlying metabolic etiology, reduce stigmatization, and improve consistency in clinical trial enrollment. MASLD diagnosis requires hepatic steatosis plus at least one cardiometabolic risk factor. Although the MASLD definition differs conceptually from traditional NAFLD criteria, multiple validation studies demonstrate high diagnostic concordance between both classification systems (8–10). Therefore, this review consistently employs MASLD terminology.

MASLD pathogenesis involves complex interactions among multiple pathological processes, including excessive fatty acid accumulation, dysregulated inflammatory responses, oxidative stress, and insulin resistance. Carnitine, an essential molecule in cellular lipid transport, may ameliorate metabolic disturbances through various pathways, including regulating fatty acid metabolism, mitigating oxidative stress, and improving mitochondrial function. While existing reviews primarily focus on carnitine-lipid metabolism relationships, comprehensive analyses detailing its molecular roles across the MASLD spectrum remain limited. This review systematically synthesizes recent evidence regarding L-carnitine's effects on lipid metabolism, insulin sensitivity, hepatocyte function, and the inflammation-fibrosis axis, while discussing potential clinical applications and future research directions.

## 2 Carnitine

Carnitine (β-hydroxy-γ-trimethylaminobutyric acid) is a quaternary ammonium compound that functions as an essential cofactor in cellular energy metabolism. Its primary physiological role involves facilitating long-chain fatty acid transport across the inner mitochondrial membrane for β-oxidation. Carnitine exists as two stereoisomers: the biologically active L-carnitine and the pharmacologically inactive D-carnitine. Humans maintain carnitine homeostasis through dietary intake and endogenous synthesis, with estimated daily requirements of 0.3–1.9 mg/kg/day (11). Approximately 75% of bodily carnitine derives from dietary sources, particularly red meat and dairy products, while the remaining 25% is synthesized endogenously (12, 13).

Endogenous carnitine biosynthesis utilizes lysine and methionine as primary substrates. Lysine provides the carbon backbone, while methionine contributes methyl groups via S-adenosylmethionine. This multi-step enzymatic process culminates with γ-butyrobetaine dioxygenase (γ-BBD) catalyzing the final conversion of γ-butyrobetaine to carnitine. As the rate-limiting enzyme, γ-BBD shows predominant expression in hepatic, renal, and cerebral tissues (14). Importantly, impaired hepatic metabolic function can compromise carnitine biosynthetic capacity, potentially leading to relative deficiency that may contribute to hepatic lipid accumulation, inflammatory activation, oxidative stress, and insulin resistance.

Cellular carnitine utilization depends on active transmembrane transport mediated by organic cation transporter 2 (OCTN2). Encoded by solute carrier family 22 member 5 (*SLC22A5*), OCTN2 represents the principal high-affinity carnitine transporter in humans, facilitating cellular uptake in intestine, kidneys, skeletal muscle, heart, and liver. *SLC22A5* mutations impair OCTN2 function, causing primary carnitine deficiency (PCD) (15–17), an autosomal recessive disorder characterized by skeletal myopathy, progressive cardiomyopathy, hypoglycemia, and hyperammonemia.

Beyond genetic determinants, OCTN2 expression and function undergo significant physiological and pharmacological regulation. Inflammatory conditions, particularly inflammatory bowel disease with elevated pro-inflammatory cytokines (TNF-α, IL-1β, IFN-γ), can suppress OCTN2 expression, reducing intestinal L-carnitine absorption (18). Additionally, certain pharmacological agents, including cisplatin, downregulate renal OCTN2 expression, increasing urinary carnitine excretion and potential deficiency states (19). Although these acquired “functional deficiencies” generally produce less severe effects than genetic mutations, they can significantly disrupt metabolic homeostasis in susceptible individuals.

Accumulating evidence indicates that appropriate L-carnitine supplementation has demonstrated beneficial effects in various pathological conditions. Documented benefits include improved insulin sensitivity in type 2 diabetes (20), reduced inflammatory cytokines and short-term mortality in sepsis (21), and ameliorated cardiac fibrosis in experimental heart failure models (22). Consequently, maintaining adequate L-carnitine levels appears crucial for hepatic lipid homeostasis and systemic energy metabolism, and may also help attenuate liver inflammation and fibrosis progression. Given that L-carnitine represents the predominant biologically active form in humans, this review focuses specifically on its mechanisms and potential applications in MASLD.

## 3 Effects of L-carnitine on systemic metabolism

### 3.1 L-carnitine promotes fatty acid metabolism

**Figure 1:**
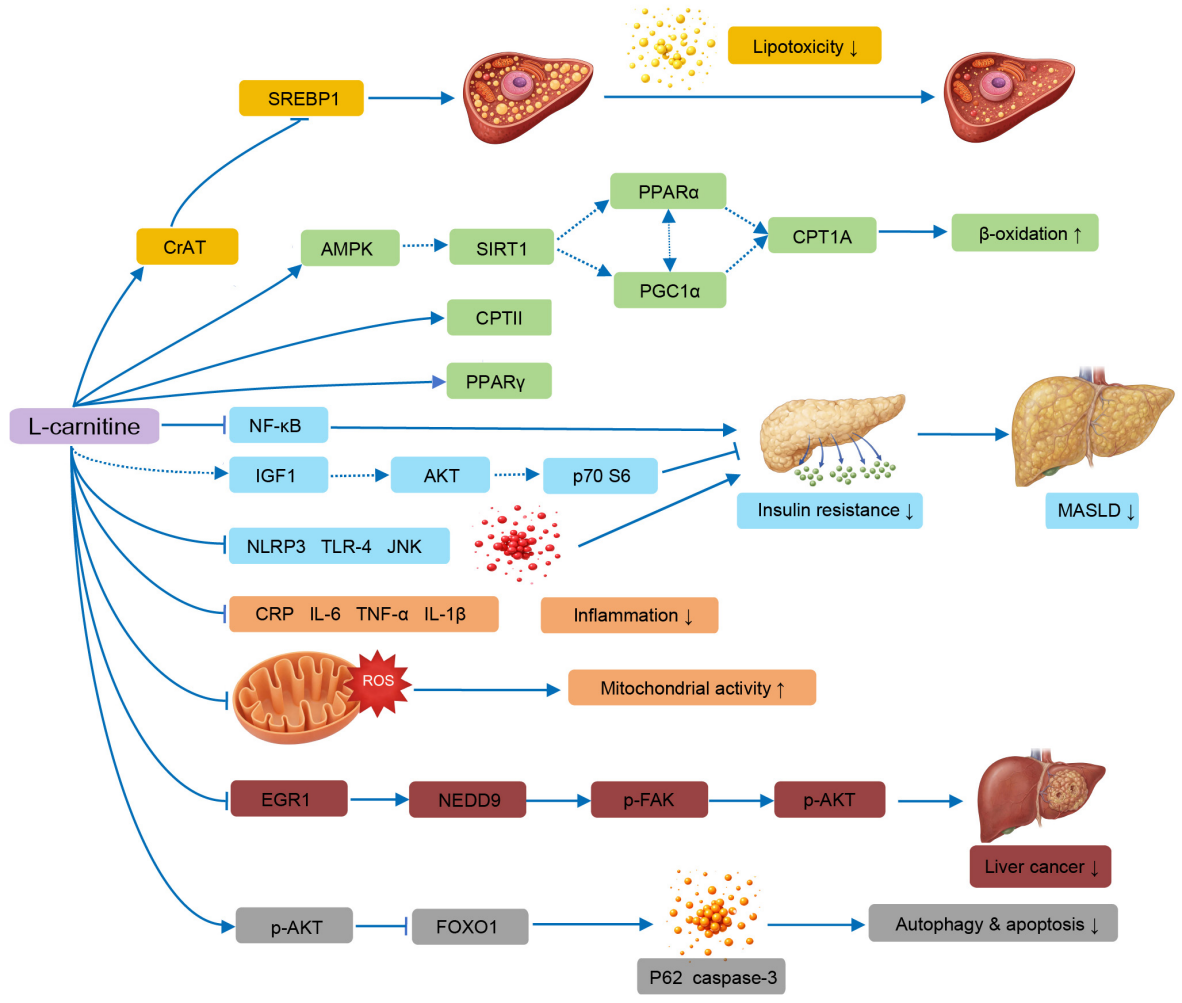
Proposed integrative mechanisms by which L-carnitine ameliorates metabolic dysfunction–associated steatotic liver disease (MASLD). L-carnitine enhances PPARα signaling and upregulates CPT1A and CPT2, thereby promoting mitochondrial fatty acid β-oxidation, improving mitochondrial activity, and reducing hepatic lipotoxicity while suppressing SREBP1-driven lipogenesis. In parallel, L-carnitine attenuates inflammatory signaling by inhibiting NF-κB activation and NLRP3 inflammasome assembly, resulting in reduced production of pro-inflammatory mediators (e.g., TNF-α, IL-6, IL-1β, CRP) and suppression of JNK/TLR-4 signaling. L-carnitine may also modulate insulin sensitivity through IGF1-associated AKT and p70S6K signaling and regulate hepatocellular stress responses via the EGR1/NEDD9/FAK/AKT axis as well as FOXO1-mediated autophagy and apoptosis. Collectively, these coordinated effects contribute to the amelioration of hepatic steatosis, inflammation, insulin resistance, and cellular stress, with potential implications for hepatocyte survival and disease progression, thereby attenuating MASLD progression. Symbols: → , activation or promotion; — → , pathway supported by limited or indirect evidence; –|, inhibition; ↑/↓, increase or decrease. Dashed arrows indicate mechanisms primarily supported by conference-level evidence or extrapolated from non–L-carnitine-specific studies and therefore require further experimental validation. Observations were derived from studies using L-carnitine alone or L-carnitine–containing complexes; the LC → CrAT → SREBP1 pathway reflects findings from LC-complex–based studies.

The multifaceted mechanisms by which L-carnitine modulates metabolic dysfunction–associated steatotic liver disease are schematically summarized in Figure 1. One of the central metabolic actions of L-carnitine is the regulation of fatty acid metabolism in hepatocytes.

Fatty acid metabolism encompasses long-chain, medium-chain, and short-chain fatty acid processing, with carnitine playing an indispensable role in long-chain fatty acid catabolism. The carnitine-dependent transport system involves coordinated enzymatic reactions. Long-chain fatty acids are first activated to acyl-CoA esters in the cytosol. Carnitine palmitoyltransferase I (CPT1), located on the outer mitochondrial membrane, then catalyzes acyl transfer from acyl-CoA to carnitine, forming acylcarnitine. This reaction represents the primary rate-limiting step in fatty acid oxidation. The resulting acylcarnitine crosses the inner mitochondrial membrane via carnitine-acylcarnitine translocase (CACT). Within the mitochondrial matrix, carnitine palmitoyltransferase II (CPT2) regenerates acyl-CoA from acylcarnitine, releasing free carnitine for recycling. The acyl-CoA undergoes sequential β-oxidation, generating acetyl-CoA that enters the tricarboxylic acid (TCA) cycle for energy production or converts to ketone bodies in hepatic mitochondria (15, 23). This elaborate transport mechanism highlights the absolute dependence of long-chain fatty acid β-oxidation on carnitine availability, contrasting with medium-chain and short-chain fatty acids that enter mitochondria independently (24, 25).

Building on these mechanistic insights, Clinical observations support carnitine's metabolic relevance in fatty liver disease, as NASH patients show significantly reduced serum L-carnitine levels compared with healthy controls (26). This carnitine deficiency appears progressive, with more pronounced reductions in patients having disease duration exceeding 5 years (27), suggesting a potential pathophysiological role in disease progression.

Preclinical investigations consistently demonstrate that L-carnitine supplementation ameliorates metabolic disturbances in experimental NAFLD/NASH models. Multiple reports indicate that L-carnitine or carnitine-containing compounds reduce serum lipids and improve liver enzyme profiles in high-fat diet (HFD)-induced rodent models (28–34). Particularly insightful mechanistic evidence comes from a medaka fish NAFLD model, where metabolomic analysis revealed that L-carnitine not only reduced hepatic lipid deposition but also significantly elevated hepatic β-oxidation end products (acetyl-CoA and ATP) while upregulating antioxidant enzyme superoxide dismutase 2 (SOD2) (35). These findings suggest a dual mechanism enhancing fatty acid catabolism while reducing oxidative stress. Preclinical evidence supporting these metabolic and regulatory effects of L-carnitine in MASLD, derived from animal and *in vitro* models, is summarized in Table 1.

**Table 1:** Animal and *in vitro* studies on L-carnitine supplementation in MASLD.

Reference	Disease	Model (Sample size)	Duration	Dose	Outcomes
Sun et al. (28)	NAFLD	Mice, 70 (60/10)	10 weeks	0.2% to 4% L-carnitine in diet	↓ TG, AST, ALT, body weight, liver weight, TNF-α, IL-6 ↑ IL-10
Mollica et al. (47)	NAFLD	Mice, 30 (10/group)	3 weeks	200 mg/kg/day	↓ lipid accumulation, ballooning ↓ROS, Cytochrome c, α-SMA, Caspase-2, NF-κB p65 ↑ PPARγ
Montesano et al. (43)	NAFLD	HepG2 cells, N/A	48 h and 72 h	5 mM L-carnitine	↓ lipid accumulation, ROS ↑ AMPK activation, PGC1α, SOD2, Nrf2
Ishikawa et al. (45)	NASH	Mice, 24 (8/group)	8 weeks	0.28% L-carnitine in diet	↓ NAS and steatosis ↓ 8-OHdG, 4-HNE, TNF-α ↑ mRNA of *Octn2, Cpt1a, Cpt2, Acadm, Sod2, Cat* ↓ liver tumorigenesis
Amer et al. (59)	NASH	Mice, 36 (6/group)	13 days	300 or 500 mg/kg/day	↓ NASH histology and serum liver enzymes ↓ hepatic oxidative stress and lipogenesis (SREBP-1) ↑ insulin signaling (increased p-Akt/Akt; decreased nuclear FOXO1) ↓ autophagy dysfunction, apoptosis, and necroptosis
Abd Eldaim et al. (30)	NAFLD	Mice, 56 (8/group)	8 weeks (preventive)/10 weeks (therapeutic)	200 mg/kg/day	↓ TC, TG, ALT, AST, Ur, Cr, MDA ↑ GSH ↓ steatosis, ballooning degeneration, lobular inflammation, and apoptosis ↓ TNF-α, TGF-β1
Lyu et al. (73)	MASH	Mice, Fibrosis:40 (10/group); HCC:102 (varies)	20, 38, and 68 weeks (terminal endpoint)	0.5% or 1% L-carnitine in diet	↓ steatosis, inflammation, and fibrosis ↓ liver tumors ↓ EGR1/NEDD9/FAK/AKT pathway

MASLD, metabolic dysfunction–associated steatotic liver disease; NAFLD, non–alcoholic fatty liver disease; NASH, non–alcoholic steatohepatitis; MASH, metabolic dysfunction–associated steatohepatitis. **Notes on nomenclature:** NAFLD and NASH correspond to MASLD and MASH, respectively, according to the current consensus.

**Biochemical and histological parameters:** ALT, alanine aminotransferase; AST, aspartate aminotransferase; GSH, glutathione; MDA, malondialdehyde; NAS, NAFLD activity score; ROS, reactive oxygen species; TC, total cholesterol; TG, triglycerides; Ur, urea.

**Proteins, cytokines, and signaling molecules (upright font):** α-SMA, α-smooth muscle actin; Caspase−2; Cytochrome c; FOXO1, forkhead box O1; IL−6, interleukin−6; IL−10, interleukin−10; NF–κB p65, nuclear factor kappa–B p65 subunit; p–Akt, phosphorylated protein kinase B; PPARγ, peroxisome proliferator–activated receptor gamma; SREBP−1, sterol regulatory element–binding protein 1; TGF–β1, transforming growth factor beta 1; TNF–α, tumor necrosis factor–alpha. **Signaling pathway components (EGR1/NEDD9/FAK/AKT):** early growth response 1, neural precursor cell expressed developmentally down–regulated 9, focal adhesion kinase, protein kinase B.

**Gene symbols (italicized)** indicate mouse transcripts.

**Symbols:** ↓, significant decrease; ↑, significant increase.

Clinical studies corroborate these experimental findings. L-carnitine supplementation improves serum lipid parameters—including total cholesterol (TC), triglycerides (TG), and low-density lipoprotein (LDL) —in NASH patients (36–38). High-dose carnitine orotate complex (Godex^®^) intervention for 6 months significantly decreased controlled attenuation parameter (CAP) values in NAFLD patients, indicating reduced hepatic steatosis (39). Body composition analyses reveal that L-carnitine treatment reduces fat mass, body weight, and body mass index (BMI) in NASH patients (40). Importantly, a randomized controlled trial demonstrated that L-carnitine supplementation reduces the intramyocellular-to-extramyocellular lipid ratio in NAFLD patients, suggesting enhanced clearance and utilization of intracellular lipid stores in skeletal muscle independent of weight changes (41).

Molecular regulation of fatty acid β-oxidation extends beyond core enzymatic machinery (CPT1, CPT2) to include sophisticated transcriptional and post-translational control mechanisms involving AMP-activated protein kinase (AMPK), peroxisome proliferator-activated receptor α (PPARα), and peroxisome proliferator-activated receptor γ coactivator-1α (PGC1α) (23, 42–44). Multiple lines of evidence indicate that L-carnitine supplementation modulates these regulatory networks in NAFLD/NASH models. Sunagozaka et al. (45) reported increased *Ppar*α gene expression in NASH mice after L-carnitine treatment, while Ishikawa et al. (46) observed concurrent upregulation of *Ppar*α and *Ppar*γ. Mollica et al. (47) demonstrated elevated Pparγ protein levels in NAFLD mice receiving L-carnitine.

AMPK serves as a central energy sensor regulating hepatic fatty acid metabolism. During fasting, AMPK activation stimulates sirtuin 1 (SIRT1), which deacetylates and activates transcriptional coactivator PGC1α (48, 49). SIRT1 also enhances PPARα transcriptional activity (50), while PGC1α functions as a PPARα coactivator to promote β-oxidation (51). Basic research indicates that both PPARα and PGC1α stimulate CPT1A transcription by binding distinct regulatory regions within the CPT1A promoter (52). Jun et al. (51) confirmed that L-carnitine increases *CPT1A* and *PPAR*γ mRNA expression in NAFLD rats. In 2021, Hong et al. (33) demonstrated that carnitine orotate complex treatment significantly enhances AMPK activation while upregulating *Sirt1, Pgc1*α*, Ppar*α*, Ucp2, and Cpt1* expression in the livers of high-fat diet-fed mice with hepatic steatosis. Complementary *in vitro* evidence from Montesano et al. (43) shows that L-carnitine reverses fructose-induced AMPK inhibition, increases PGC1α protein levels, reduces reactive oxygen species (ROS) production, and enhances antioxidant factor expression in HepG2 cells. Collectively, these findings suggest that L-carnitine promotes β-oxidation, reduces hepatic lipid accumulation, and exerts antioxidant effects, potentially through the AMPK-SIRT1-PPAR-PGC1α-CPT1 signaling network. Evidence for each component of this network comes from separate preclinical studies, and the precise mechanisms in human MASLD remain to be fully elucidated.

L-carnitine also modulates hepatic lipogenesis through sterol regulatory element-binding protein 1 (SREBP1) regulation. In HFD-fed KK-Ay mice, L-carnitine administration inhibited *Srebp1c* mRNA overexpression while increasing β-hydroxybutyrate levels, suggesting concurrent lipogenesis suppression and β-oxidation activation (29). Hong et al. (33) provided mechanistic depth by demonstrating that carnitine orotate complex increases carnitine acetyltransferase (CrAT) and β-oxidation-related proteins (CPT1, PPARα, UCP2) while inhibiting SREBP1c nuclear translocation and downstream target gene expression (*Hmgcr, Srebp2, Pcsk9, Ldlr*) in HFD-fed mice. Crucially, *Crat* knockout experiments partially reversed these metabolic improvements, establishing CrAT-mediated SREBP1c inhibition as a key mechanism through which L-carnitine counteracts *de novo* lipogenesis. In summary, L-carnitine appears to balance hepatic lipid metabolism by simultaneously promoting β-oxidation and inhibiting lipogenesis, though detailed SREBP1-related pathway mechanisms warrant further investigation.

At the translational clinical level, L-carnitine primarily targets hepatic steatosis—the earliest pathological step in MASLD. Evidence indicates that supplementation (typically 2–4 g/day) significantly reduces liver fat content and improves systemic lipid profiles. Future translational research should aim to define the “carnitine-responsive” phenotype using integrated approaches, including serum acylcarnitine profiling, quantitative MRI of muscle fat, and *CPT1A* genotyping to enable precise patient stratification. Building on this, its role as a “metabolic booster” alongside lifestyle interventions, or sequentially with GLP-1 receptor agonists to prevent hepatic fat re-accumulation, warrants further investigation. Additionally, developing novel efficacy endpoints beyond conventional CAP, such as evaluating the specific modulation of hepatic triglyceride composition—particularly the proportion of lipotoxic saturated fatty acids—may more directly elucidate the mechanisms underlying its hepatoprotective effects.

### 3.2 L-carnitine promotes glucose metabolism and improves insulin resistance

Insulin resistance (IR) represents a fundamental pathogenic mechanism in MASLD, operating throughout the disease spectrum from simple steatosis to MASH and progressive liver fibrosis. L-carnitine's potential to modulate impaired glucose metabolism in MASLD has attracted increasing investigative attention. Clinical evidence demonstrates that L-carnitine supplementation reduces fasting blood glucose (37, 39, 53, 54), insulin levels (54, 55), glycated hemoglobin (HbA1c) (56, 57), and ameliorates insulin resistance (37, 53, 54) in NAFLD patients. A 2020 meta-analysis of five randomized controlled trials (RCTs) encompassing 334 NAFLD patients confirmed that L-carnitine supplementation significantly improves insulin sensitivity (58). Preclinical studies generally support these clinical observations. NASH model mice treated with L-carnitine exhibited significant improvements in hyperglycemia and hyperinsulinemia (29, 59). Godex^®^ markedly reduced fasting blood glucose and insulin resistance indices while enhancing insulin sensitivity in NAFLD mice (33). However, not all interventions proved effective, as acetyl-L-carnitine plus alpha-lipoic acid failed to reverse insulin resistance in another NAFLD mouse model (32). Overall, L-carnitine may improve insulin resistance in MASLD, with effects influenced by formulation, dosage, treatment length, and experimental model.

Mechanistic insights into L-carnitine's effects on insulin resistance include:

Inhibition of NF-κB-mediated chronic inflammation: NF-κB signaling promotes systemic inflammation and directly interferes with insulin signaling pathways, exacerbating IR (60). L-carnitine suppressed NF-κB expression and downregulated key inflammatory mediators, including NLRP3 inflammasome, Toll-like receptor 4 (TLR4), and c-Jun N-terminal kinase (JNK) (28).

Synergistic upregulation of energy metabolism and insulin signaling genes: Salic et al. (61) demonstrated that combined L-carnitine and nicotinamide riboside administration upregulated multiple metabolic and insulin signaling regulators (*Ppargc1b, Acox1*), promoting mitochondrial oxidative phosphorylation and peripheral glucose uptake.

*In vitro* evidence suggests that L-carnitine may modulate insulin-like signaling pathways in hepatocytes, including IGF1-associated AKT and p70 S6 kinase signaling; however, this observation is primarily supported by conference-level data and requires further validation (62, 63).

While these findings provide mechanistic plausibility for L-carnitine's effects on IR, most evidence derives from animal or cellular models, with limited human histological validation or pathway-intervention studies. Future multicenter, large-sample RCTs incorporating liver transcriptomics and metabolomics are warranted to optimize dosing and treatment duration in MASLD patients with IR.

The clinical significance of L-carnitine in ameliorating insulin resistance (IR) lies in its intervention in a core pathogenic feature of MASLD: hepatic IR. Meta-analyses of randomized controlled trials indicate that supplementation significantly improves insulin sensitivity, as reflected by reduced HOMA-IR. Future translational efforts should pursue several directions: first, conducting deep-phenotyping trials in biopsy-confirmed MASH patients, using positron emission tomography to quantify hepatic glucose uptake and correlating these findings with intrahepatic insulin signaling (e.g., p-AKT). Second, positioning L-carnitine as a “metabolic re-sensitizer” to evaluate its potential, alone or combined with SGLT2 inhibitors, in restoring hepatic insulin sensitivity among type 2 diabetes patients with MASLD who respond poorly to or cannot tolerate metformin. Finally, delineating tissue-preferential effects is essential to determine whether improvements in IR arise primarily from reduced hepatic lipid content or also involve amelioration of pancreatic steatosis and skeletal muscle lipid metabolism.

## 4 Regulatory mechanisms of L-carnitine on hepatocyte function

Clinical evidence indicates that L-carnitine improves liver function in NAFLD patients, reflected by reduced serum alanine aminotransferase (ALT) and aspartate aminotransferase (AST) levels (37, 63). L-carnitine-containing compounds demonstrate comparable hepatoprotective effects. One RCT reported that L-carnitine co-administered with magnesium hydroxide significantly reduced serum AST and ALT (64), while another study found combined L-carnitine and resistance training superior to either intervention alone for liver enzyme reduction (65). In diabetic NAFLD populations, Godex^®^ treatment for 12 weeks significantly improved liver enzymes (56), and L-carnitine monotherapy for 3 months similarly enhanced hepatic function in type 2 diabetes patients with NAFLD (66). Notably, a 2014 randomized double-blind study by Hong et al. demonstrated that adding carnitine orotate complex (900 mg/day, 12 weeks) to metformin therapy significantly augmented ALT reduction versus metformin alone (67). However, the therapeutic response may not be uniform across all patient populations. Age may influence therapeutic efficacy, as a meta-analysis by Liu et al. (36) found no significant liver enzyme improvement in pediatric patients, primarily based on a single pediatric study that itself showed no clear benefit (68). Mitochondrial dysfunction and oxidative stress constitute central mechanisms in MASH pathogenesis, with MASLD progression closely linked to mitochondrial impairment (69). Consequently, mitochondrial restoration represents a promising therapeutic target in MASLD (70, 71). L-carnitine exerts hepatoprotective effects primarily through mitochondrial mechanisms. Clinical observations by Hong et al. revealed that L-carnitine supplementation increased peripheral blood mitochondrial DNA (mtDNA) copy number while decreasing urinary 8-hydroxy2'-deoxyguanosine (8-OHdG) levels (67), providing clinical evidence of improved mitochondrial function and reduced oxidative stress. Basic research supports this mechanism, demonstrating that fructose or HFD exposure induces mitochondrial structural abnormalities, mtDNA depletion, and ATP deficiency, ultimately impairing β-oxidation and hepatocyte function. L-carnitine supplementation mitigated these mitochondrial alterations, restored mtDNA levels, and enhanced β-oxidation capacity (29, 33, 43, 72, 73).

Human studies further corroborate these findings. In NAFLD patients, Godex^®^ supplementation significantly reduced ALT and AST, increased mtDNA copy number, and decreased oxidative stress marker 8-oxo-7, 8-dihydro2'-deoxyguanosine (8-oxo-dG) (74). Analysis of 14 paired liver biopsies revealed significantly lower baseline peripheral mtDNA copy number in NASH versus simple steatosis patients, establishing an association between mitochondrial dysfunction and disease severity.

L-carnitine also ameliorates oxidative stress by reducing ROS production (43, 47, 75), decreasing hepatic malondialdehyde (MDA) content (30, 59, 76), lowering 8-OHdG concentrations, and attenuating 4-hydroxynonenal (4-HNE) immunostaining intensity (45). Concurrently, it enhances antioxidant defenses through SOD2 and nuclear factor erythroid 2-related factor 2 (Nrf2) upregulation (43), ameliorating hepatic inflammation in NAFLD models. Furthermore, L-carnitine upregulates genes involved in fatty acid transport and mitochondrial β-oxidation (*Octn2, Cpt1a, Cpt2, Acadm*) while increasing metabolites associated with TCA cycle, urea cycle, and antioxidant pathways (malate, citrulline) (45). These coordinated effects collectively promote hepatic metabolic homeostasis restoration.

Beyond mitochondrial mechanisms, L-carnitine inhibits hepatocyte autophagy and apoptosis. In HepG2 cells, L-carnitine reduced free fatty acid-induced apoptosis (72), while similar anti-apoptotic effects occurred in oleic acid-treated hepatocytes (77). A 2024 study in dexamethasone-induced NASH rats demonstrated that L-carnitine restored Akt phosphorylation, inhibited FOXO1 nuclear translocation, and downregulated autophagy and apoptosis markers (LC3, P62, caspase-3, p-MLKL) via the Akt/FOXO1 pathway (59). In summary, L-carnitine improves MASLD through multiple mechanisms including mitochondrial protection, oxidative stress reduction, and autophagy/apoptosis suppression. While current research focuses predominantly on hepatocytes, potential Kupffer cell modulation in NASH-related inflammation and fibrosis warrants further investigation (78).

The hepatoprotective properties of L-carnitine confer clinical value beyond mere transaminase reduction, potentially delaying disease progression by stabilizing mitochondrial function. Clinically observed concomitant decreases in serum ALT/AST and increases in peripheral blood mitochondrial DNA copy number highlight its *in vivo* bioactivity. To advance translational applications, future studies should focus on: (1) transforming dynamic biomarkers into clinical decision-making tools, such as evaluating early changes in circulating mitochondrial-derived vesicles or oxidative stress markers (e.g., 8–iso–prostaglandin F2α (8-iso-PGF2α))to predict subsequent biochemical and histological responses; (2) developing combination therapies for specific pathological subtypes, including potential synergistic benefits with mitochondria-targeted antioxidants (e.g., MitoQ) or bile acid modulators (e.g., ursodeoxycholic acid, UDCA) in MASH patients with prominent ballooning degeneration or cholestatic features; and (3) assessing prophylactic hepatoprotective potential in high-risk MASLD populations, such as patients on concomitant statin therapy, to broaden clinical applicability.

## 5 Mechanisms of the anti-inflammatory and anti-fibrotic effects of L-carnitine

Preclinical studies consistently demonstrate that L-carnitine ameliorates liver pathology in NAFLD animal models, improving steatosis, lobular inflammation, and fibrosis (30, 31, 45, 47, 72, 73). L-carnitine-containing compounds show comparable efficacy (33, 34, 61, 79, 80). The anti-inflammatory effects involve inflammatory mediator and signaling pathway suppression. NAFLD-associated chronic low-grade inflammation responds to L-carnitine supplementation through reduced hepatic pro-inflammatory cytokines (e.g., IL-6), increased anti-inflammatory cytokine IL-10, and downregulation of key inflammatory pathway components (TLR4, NF-κB, JNK) (28).

NF-κB represents a master regulator of inflammatory responses in NAFLD progression. Ligand-activated PPARγ can inhibit NF-κB signaling by impeding p65 nuclear translocation and reducing p65/PPARγ complex formation (81). In methionine-choline deficient (MCD) diet-induced NAFLD mice, L-carnitine upregulated hepatic PPARγ protein expression while inhibiting NF-κB p65 activation, thereby attenuating liver inflammation and fibrosis (47). Similarly, in HFD-induced obese rats, L-carnitine reduced TNF-α and TGF-β1 protein levels, further mitigating inflammatory responses (30). Cellular studies confirm TNF-α, TGF-β1, and procollagen type I mRNA downregulation following L-carnitine treatment (77).

Clinical evidence supports these anti-inflammatory effects. Malaguarnera (37), Amiri-Moghadam (82), and Zvyagintseva et al. (27) reported reduced serum CRP, TNF-α, and IL-6 levels in NASH patients after L-carnitine intervention. The randomized controlled trial by Malaguarnera et al. showed that 24-week L-carnitine treatment improved liver histology, including reduced lobular inflammation, portal inflammation, and fibrosis (37). A 2024 study in MASH patients and mouse models further confirmed that L-carnitine supplementation suppresses inflammatory gene expression (*Tnf, Il6, Il1b*) and downregulates pro-fibrotic genes (*Acta2, Col1a2, Tgfb1, Pdgfrb, Pdgf-b, Pdgf-c*), reducing α-smooth muscle actin (α-SMA) and platelet-derived growth factor receptor beta (PDGFRβ) protein levels (73). Collectively, these preclinical and clinical findings indicate that L-carnitine can delay MASLD progression by mitigating hepatic inflammation and fibrosis through multiple interconnected mechanisms.

The anti-inflammatory and anti-fibrotic properties of L-carnitine underscore its potential to halt MASLD progression to advanced stages. Key randomized controlled trials have demonstrated its efficacy in improving histologic measures of hepatic inflammation and fibrosis. Future translational strategies should prioritize two directions. First, identifying and targeting patients with an “active inflammatory-fibrotic” phenotype, typically characterized by elevated baseline IL-6 or high-sensitivity C-reactive protein, or by MRI-PDFF-evidenced steatosis with elevated T1 mapping values. Second, evaluating L-carnitine as a “background therapy” in combination with novel anti-fibrotic agents (e.g., GLP-1/GIP dual receptor agonists, FGF21 analogs) to assess its role in consolidating therapeutic efficacy and reducing relapse risk after drug discontinuation.

## 6 Tumor-suppressive properties of L-carnitine

Overall, preclinical evidence suggests that L-carnitine may contribute to hepatoprotection, although robust clinical evidence for anti-tumor efficacy in humans is currently lacking. In a novel NASH mouse model, low carnitine levels combined with impaired glucose tolerance were associated with enhanced hepatocarcinogenesis (83). Neural precursor cell expressed, developmentally down–regulated 9 (NEDD9), a scaffolding protein associated with poor prognosis in multiple cancers, shows significantly elevated expression in hepatocellular carcinoma (HCC) tissues versus adjacent non-tumor liver (84). Lyu et al. (73) reported that in hepatocytes, L-carnitine inhibited stress-induced transcription factor early growth response protein 1 (EGR1), subsequently downregulating its target gene *NEDD9* and modulating the NEDD9/FAK/AKT pathway, signaling pathway, indicating a potential protective mechanism in that experimental context.

Furthermore, L-carnitine may function as an endogenous HDAC inhibitor, promoting p21∧Cip1 expression. Huang et al. demonstrated that in HepG2 cells, L-carnitine acts as an endogenous histone deacetylase inhibitor, promoting p21∧Cip1 expression (85). This epigenetic regulation may represent another avenue for hepatoprotective effects; however, its relevance in human MASLD or metabolic HCC remains to be established.

In addition, a rat hepatitis-HCC model suggested that L-carnitine might mitigate processes associated with hepatocarcinogenesis, such as oxidative stress, and improve mitochondrial function (75). Given that chronic hepatitis and cirrhosis are major risk factors for HCC, the anti-inflammatory and anti-fibrotic effects of L-carnitine could theoretically contribute to reduced HCC risk in patients with advanced MASLD, but this hypothesis awaits clinical validation.

The potential tumor-suppressive effects of L-carnitine provide a unique chemopreventive perspective for long-term MASLD management. Although direct clinical evidence for anti-HCC activity in humans is lacking, its ability to attenuate insulin resistance, oxidative stress, and the inflammation-fibrosis cascade may indirectly reduce HCC risk by optimizing the hepatic oncogenic microenvironment. Future research should employ pragmatic and innovative strategies: first, leveraging real-world data through nested case-control studies linking dietary supplement usage with cancer registries to generate preliminary epidemiological insights into the association between long-term L-carnitine use and HCC incidence in MASLD populations; second, focusing on high-risk cohorts, such as patients with MASH-related compensated cirrhosis, using dynamic contrast-enhanced ultrasound (DCE-US) to monitor *de novo* nodule emergence and evolution (e.g., arterial phase hyperenhancement) as surrogate endpoints for preliminary chemopreventive assessment; and third, exploring potential synergy with existing HCC surveillance by investigating whether L-carnitine supplementation enhances the sensitivity of serum biomarkers such as protein induced by vitamin K absence-II (PIVKA-II) or alpha-fetoprotein L3 (AFP-L3) for early detection.

## 7 Discussion

Current MASLD management primarily relies on lifestyle interventions, including weight reduction, dietary modification, and physical activity (86, 87). However, effective pharmacotherapeutic options remain limited. The 2024 FDA approval of resmetirom, the first targeted therapy for MASLD/MASH, represents a significant advancement (88). As a thyroid hormone receptor β agonist, resmetirom improves hepatic steatosis, inflammation, and fibrosis while providing lipid and cardiovascular benefits. Nevertheless, interindividual response variability, high cost, and adherence challenges may limit widespread clinical implementation.

This review has systematically summarized evidence suggesting that L-carnitine exerts multi-targeted actions against MASLD pathogenesis by promoting lipid metabolism, improving insulin sensitivity, stabilizing hepatocyte function, and inhibiting inflammatory and fibrotic signaling pathways. As a dietary supplement, L-carnitine offers advantages including favorable safety profile, convenient administration, and potential efficacy, positioning it as a promising adjunctive option for MASLD management. Available clinical evidence regarding L-carnitine supplementation in MASLD is summarized in Table 2, highlighting both reported metabolic benefits and the current limitations and heterogeneity of human studies. Several important questions remain unresolved, including dosing regimen standardization, limited robust clinical evidence, and insufficient carnitine derivative investigation. Notably, the enantiomer D-carnitine may exert hepatotoxic effects (89), underscoring the critical importance of compound-specific safety evaluation and reinforcing that only the biologically active L-isomer should be considered for therapeutic use in MASLD.

**Table 2:** Human studies on L-carnitine supplementation in MASLD.

Reference	Disease	Sample size	Duration	Dose	Outcomes
Hamza et al. (53)	NAFLD	100 (50/50)	24 weeks	50 mg/kg/day	↓ Chemerin, BMI, WC, HC, WHR, ALT, AST, FPG, HOMA-IR, NAFLD severity ↑ Glucose/insulin ratio → TC, TG, LDL, HDL
Lyu et al. (73)	MASH	11	10 weeks	2,000 mg/day	↓ ALT, AST, GGT, TG ↓ NAS, inflammation, ballooning ↑ Oxidative stress repair (*PRDX2*) and lipid metabolism genes (*PPARA, CPT1A*) ↓ Inflammation (*CCL21*), fibrosis (*COL1A1, COL1A2*) genes → TC, FBG, HbA1c; hepatic steatosis grade, fibrosis stage
Malaguarnera et al. (37)	NASH	74 (36/38)	24 weeks	2,000 mg/day	↓ AST, ALT, GGT, TC, LDL-C, TG, FPG, HOMA-IR, CRP, TNF-α ↑ HDL-C ↓ steatosis, inflammation, ballooning, and fibrosis
Somi et al. (63)	NAFLD	80 (40/40)	24 weeks	500 mg/day	↓ AST, ALT, BMI, Weight, Sonographic grade within-group
Thiagarajan et al. (41)	NAFLD	18 (9/9)	24 weeks	4,000 mg/day	↓ ALT, IHTG, Muscle Lipid Fractions → M, Adipo-IR, body weight
Mohammadi et al. (65)	NAFLD	40 (10/group)	12 weeks	10 mg/kg/day	↓ AST, ALT, BMI, PBF in exercise and combined groups → BMI or PBF with L-carnitine supplementation alone
Alavinejad et al. (66)	NAFLD + Diabetes	60 (30/group)	3 months	750 mg/day	↓ AST, ALT → TC, TG, FBS, sonographic degree of fatty liver.

MASLD, metabolic dysfunction–associated steatotic liver disease; NAFLD, non–alcoholic fatty liver disease; NASH, non–alcoholic steatohepatitis; MASH, metabolic dysfunction–associated steatohepatitis.

**Clinical and metabolic parameters:** Adipo–IR, adipose tissue insulin resistance index; ALT, alanine aminotransferase; AST, aspartate aminotransferase; BMI, body mass index; CRP, C–reactive protein; FBG, fasting blood glucose; FPG, fasting plasma glucose; GGT, γ-glutamyl transferase; HbA1c, glycated hemoglobin; HC, hip circumference; HDL, high–density lipoprotein; HOMA–IR, homeostatic model assessment of insulin resistance; IHTG, intrahepatic triglyceride content; LDL, low–density lipoprotein; M, whole–body insulin sensitivity (hyperinsulinemic–euglycemic clamp); NAS, NAFLD activity score; PBF, percentage of body fat; TC, total cholesterol; TG, triglycerides; WC, waist circumference; WHR, waist–to–hip ratio.

**Gene symbols (italicized)** indicate human transcripts (e.g., *PRDX2, PPARA*).

**Symbols:** ↓, significant decrease; ↑, significant increase; → , no significant change.

Recent research has illuminated the gut-liver axis in MASLD pathogenesis (90, 91). One study reported that a metabolic cofactor combination (L-carnitine, betaine, N-acetylcysteine, nicotinamide riboside) improved intestinal architecture, reduced permeability, and restored tight junction protein expression in NAFLD mice, thereby enhancing gut barrier function (92). Gut inflammasome activation shows close NAFLD association (93), and combined L-carnitine and vitamin E supplementation upregulated gut inflammasome-related genes while ameliorating liver inflammation and fibrosis in a NASH mouse model (94). These findings suggest that gut-liver axis modulation may contribute to L-carnitine's mechanisms of action, although the precise molecular mechanisms remain to be elucidated.

Animal model metabolomics studies reveal that HFD-induced NAFLD is characterized by hepatic saturated fatty acid (myristic acid, palmitic acid) and monounsaturated fatty acid (oleic acid) accumulation, accompanied by decreased gluconeogenic amino acid (glycine, alanine, aspartic acid, glutamic acid, proline) levels (95). However, the specific role of carnitine, a key molecule involved in lipid metabolism, in these metabolic alterations remains inadequately explored.

Optimal L-carnitine dosing requires further clarification. An early meta-analysis suggested that 3 g/day may be necessary to normalize serum ALT in a significant patient proportion (96), while other studies report benefits with 2 g/day on AST, ALT, and HOMA-IR (37). Bae et al. (56) demonstrated that Godex^®^ at 900 mg/day improved AST, ALT, and HbA1c in diabetic NAFLD patients. These variable effective doses highlight the need for systematic dose-response studies to establish optimal dosing regimens.

Beyond dose optimization, combining L-carnitine with nutrients or drugs may synergistically enhance its multi-target metabolic effects by regulating mitochondrial function, fatty acid oxidation, and insulin signaling. For example, co-administration of L-carnitine and α-lipoic acid did not improve insulin resistance but markedly ameliorated mitochondrial ultrastructure and reduced serum liver enzyme levels, suggesting a preferential correction of mitochondrial injury (32). In Ldlr^−^/^−^Leiden mice, only the combined intervention with nicotinamide riboside, rather than either monotherapy, significantly attenuated obesity and hepatic steatosis, accompanied by coordinated upregulation of genes involved in fatty acid oxidation and insulin signaling (61). Mechanistically, the metabolic benefits of Godex^®^ depend on enhanced activity of CrAT, which modulates mitochondrial acetyl-CoA metabolism and thereby improves insulin sensitivity and hepatic lipid homeostasis (33). However, these combination effects are highly dependent on experimental models and co-administered components: synergistic metabolic improvements have been observed in animal studies (80), whereas some human studies report only modest reductions in liver enzymes without significant changes in broader metabolic parameters (64). Clinically, accumulating evidence indicates that combinations of L-carnitine with vitamin E, silymarin, or simvastatin can improve liver function and selected metabolic indices (38, 54, 55). Overall, the therapeutic potential of L-carnitine–based combination strategies is most likely to be maximized when they are tailored to specific pathological contexts and concurrently target key processes such as fatty acid oxidation, insulin signaling, oxidative stress, and gut barrier integrity (92). These findings highlight the potential of combination strategies to maximize metabolic benefits, warranting further investigation in clinical studies.

Building on these findings, the clinical evidence collated in Table 2 reveals substantial heterogeneity in the therapeutic efficacy of L-carnitine supplementation for MASLD. Although certain trials demonstrate benefits in insulin sensitivity and hepatic steatosis, others report neutral outcomes. This discrepancy primarily stems from methodological variations in patient selection, intervention design, and endpoint assessment, rather than contradicting the established biological plausibility of LC's mechanisms. Patient phenotype critically defines the responsive subset. Individuals with active MASH—marked by significant mitochondrial dysfunction and inflammatory activity—represent a more susceptible population. Here, LC, functioning as an essential cofactor and metabolic regulator, directly counteracts core pathological processes by enhancing mitochondrial β-oxidation and attenuating oxidative stress. In contrast, patients with early-stage steatosis or those embedded in a context of profound systemic insulin resistance may present a less amenable target for short-term LC monotherapy, as the driving pathophysiology extends beyond immediate fatty acid flux. Pharmacological determinants—dose and duration—govern target engagement. Higher doses (≥2000 mg/day) likely saturate hepatic OCTN2 transporters and elevate intracellular carnitine sufficiently to activate CPT1-mediated fatty acid oxidation. Intervention duration influences the cumulative process from molecular adaptations to tissue-level pathological improvements. Quantitative endpoints, such as MRI-PDFF and histology, can capture subtle yet clinically meaningful changes, whereas ALT/AST and ultrasonography may miss early metabolic improvements. Together, these findings underscore that LC's variable clinical efficacy arises from interacting factors including patient phenotype, dosing strategy, intervention duration, and endpoint sensitivity.

In conclusion, MASLD pathogenesis involves complex interactions among multiple metabolic pathways, including dysregulated lipid metabolism, insulin resistance, inflammation, and oxidative stress. L-carnitine, as a key cofactor in fatty acid transport and energy metabolism, exerts multifaceted effects that may modulate these pathogenic processes. While preclinical studies provide promising evidence, clinical validation remains limited across disease stages. Future research should focus on elucidating L-carnitine's molecular mechanisms within the hepatic metabolic network and rigorously assessing its safety and efficacy in well-designed clinical trials. Such efforts hold the potential to advance novel prevention and therapeutic strategies for MASLD.

Previous reviews have summarized the biological functions and therapeutic potential of L-carnitine primarily within the traditional NAFLD/NASH framework (97, 98), but they have not systematically reassessed its relevance under the updated diagnostic criteria and conceptual transition to MASLD. Moreover, emerging mechanistic and clinical evidence published in recent years has not yet been comprehensively integrated. To address these gaps and advance the field, the present review provides several distinct contributions. First, we adopt the latest MASLD/MASH nomenclature to align our discussion with current international consensus. Second, we integrate recent molecular evidence spanning both classical and emerging pathways in lipid metabolism (including AMPK, SIRT1, PPARα, and PGC1α) as well as metabolic–inflammatory signaling related to insulin resistance (e.g., activation of IGF-1/AKT and inhibition of NF-κB). We also summarize newly proposed mechanisms associated with hepatoprotection and tumor suppression, such as attenuation of hepatocellular stress through inhibition of the NEDD9/FAK/AKT cascade and a potential role in cell-cycle regulation via the HDAC/p21 axis—although the latter remains to be validated in MASLD-specific models. Third, we synthesize key preclinical studies and randomized controlled trials published in the last 10 years to provide a timely and critical appraisal of efficacy and safety. Finally, we highlight major limitations in the current evidence—such as heterogeneous dosing regimens, the scarcity of long-term clinical data, and insufficient mechanistic validation under MASLD conditions—and propose directions for future translational research.

## References

[1] Younossi ZM Golabi P Paik JM Henry A Van Dongen C Henry L. The global epidemiology of nonalcoholic fatty liver disease (NAFLD) and nonalcoholic steatohepatitis (NASH): a systematic review. Hepatology. (2023) 77:1335–47. doi: 10.1097/HEP.0000000000000004 36626630 PMC10026948

[2] Singh S Allen AM Wang Z Prokop LJ Murad MH Loomba R. Fibrosis progression in nonalcoholic fatty liver vs nonalcoholic steatohepatitis: a systematic review and meta-analysis of paired-biopsy studies. Clin Gastroenterol Hepatol. (2015) 13:643-54.e1-9; quiz e39-40. doi: 10.1016/j.cgh.2014.04.014 24768810 PMC4208976

[3] Kalligeros M Henry L Younossi ZM. Metabolic dysfunction-associated steatotic liver disease and its link to cancer. Metabolism. (2024) 160:156004. doi: 10.1016/j.metabol.2024.156004 39182603

[4] Ballestri S Zona S Targher G Romagnoli D Baldelli E Nascimbeni F. et al. Nonalcoholic fatty liver disease is associated with an almost twofold increased risk of incident type 2 diabetes and metabolic syndrome Evidence from a systematic review and meta-analysis. J Gastroenterol Hepatol. (2016) 31:936–44. doi: 10.1111/jgh.13264 26667191

[5] Abosheaishaa H Hussein M Ghallab M Abdelhamid M Balassiano N Ahammed MR. et al. Association between non-alcoholic fatty liver disease and coronary artery disease outcomes: A systematic review and meta-analysis Diabetes. Metab Syndr. (2024) 18:102938. doi: 10.1016/j.dsx.2023.102938 38194827

[6] Mantovani A Petracca G Beatrice G Csermely A Lonardo A Schattenberg JM. et al. Non-alcoholic fatty liver disease and risk of incident chronic kidney disease: an updated meta-analysis. Gut. (2022) 71:156–62. doi: 10.1136/gutjnl-2020-323082 33303564

[7] Rinella ME Lazarus JV Ratziu V Francque SM Sanyal AJ Kanwal F. et al. A multisociety Delphi consensus statement on new fatty liver disease nomenclature. J Hepatol. (2023) 79:1542–56. doi: 10.1097/HEP.0000000000000696 37364790

[8] Song SJ Lai JC Wong GL Wong VW Yip TC. Can we use old NAFLD data under the new MASLD definition? J Hepatol. (2024) 80:e54–e6. doi: 10.1016/j.jhep.2023.07.021 37541393

[9] Ratziu V Boursier J. Confirmatory biomarker diagnostic studies are not needed when transitioning from NAFLD to MASLD. J Hepatol. (2024) 80:e51–e2. doi: 10.1016/j.jhep.2023.07.017 37543307

[10] Hagström H Vessby J Ekstedt M Shang Y. 99% of patients with NAFLD meet MASLD criteria and natural history is therefore identical. J Hepatol. (2024) 80:e76–e7. doi: 10.1016/j.jhep.2023.08.026 37678723

[11] Demarquoy J Georges B Rigault C Royer MC Clairet A Soty M. et al. Radioisotopic determination of L-carnitine content in foods commonly eaten in Western countries. Food Chem. (2004) 86:137–42. doi: 10.1016/j.foodchem.2003.09.023

[12] Steiber A Kerner J Hoppel CL. Carnitine: a nutritional, biosynthetic, and functional perspective. Mol Aspects Med. (2004) 25:455–73. doi: 10.1016/j.mam.2004.06.006 15363636

[13] Pekala J Patkowska-Sokoła B Bodkowski R Jamroz D Nowakowski P Lochyński S. et al. L-carnitine–metabolic functions and meaning in humans life. Curr Drug Metab. (2011) 12:667–78. doi: 10.2174/138920011796504536 21561431

[14] Vaz FM Wanders RJ. Carnitine biosynthesis in mammals. Biochem J. (2002) 361:417–29. doi: 10.1042/bj3610417 11802770 PMC1222323

[15] Longo N Amat di San Filippo C Pasquali M. Disorders of carnitine transport and the carnitine cycle. Am J Med Genet C Semin Med Genet. (2006) 142c:77-85. doi: 10.1002/ajmg.c.30087 16602102 PMC2557099

[16] Cederbaum SD Koo-McCoy S Tein I Hsu BY Ganguly A Vilain E. et al. Carnitine membrane transporter deficiency: a long-term follow up and OCTN2 mutation in the first documented case of primary carnitine deficiency. Mol Genet Metab. (2002) 77:195–201. doi: 10.1016/S1096-7192(02)00169-5 12409266

[17] Crefcoeur LL Visser G Ferdinandusse S Wijburg FA Langeveld M Sjouke B. Clinical characteristics of primary carnitine deficiency: a structured review using a case-by-case approach. J Inherit Metab Dis. (2022) 45:386–405. doi: 10.1002/jimd.12475 34997761 PMC9305179

[18] Lancaster CS Hu C Franke RM Filipski KK Orwick SJ Chen Z. et al. Cisplatin-induced downregulation of OCTN2 affects carnitine wasting. Clin Cancer Res. (2010) 16:4789–99. doi: 10.1158/1078-0432.CCR-10-1239 20858838 PMC3531239

[19] Li P Wang Y Luo J Zeng Q Wang M Bai M. et al. Downregulation of OCTN2 by cytokines plays an important role in the progression of inflammatory bowel disease. Biochem Pharmacol. (2020) 178:114115. doi: 10.1016/j.bcp.2020.114115 32579962

[20] Op den. Kamp-Bruls YMH, Op den Kamp YJM, Veeraiah P, Zapata Perez R, Phielix E, Havekes B, et al. Carnitine supplementation improves insulin sensitivity and skeletal muscle acetylcarnitine formation in patients with type 2 diabetes. Diabetes Obes Metab. (2025) 27:2864–77. doi: 10.1111/dom.16298 40019115 PMC11965010

[21] Keshani M Alikiaii B Babaei Z Askari G Heidari Z Sharma M. et al. The effects of L-carnitine supplementation on inflammation, oxidative stress, and clinical outcomes in critically Ill patients with sepsis: a randomized, double-blind, controlled trial. Nutr J. (2024) 23:31. doi: 10.1186/s12937-024-00934-4 38444016 PMC10916166

[22] Omori Y Ohtani T Sakata Y Mano T Takeda Y Tamaki S. et al. L-Carnitine prevents the development of ventricular fibrosis and heart failure with preserved ejection fraction in hypertensive heart disease. J Hypertens. (2012) 30:1834–44. doi: 10.1097/HJH.0b013e3283569c5a 22796714

[23] Kerner J Hoppel C. Fatty acid import into mitochondria. Biochim Biophys Acta. (2000) 1486:1–17. doi: 10.1016/S1388-1981(00)00044-5 10856709

[24] Schönfeld P Wojtczak L. Short- and medium-chain fatty acids in energy metabolism: the cellular perspective. J Lipid Res. (2016) 57:943–54. doi: 10.1194/jlr.R067629 27080715 PMC4878196

[25] Aas M Bremer J. Short-chain fatty acid activation in rat liver. A new assay procedure for the enzymes and studies on their intracellular localization. Biochim Biophys Acta. (1968) 164:157–66. doi: 10.1016/0005-2760(68)90142-2 5721019

[26] Franco AJ Castañé H Gaya GB Rodríguez-Tomàs E Andreu JC Maried JJ. Carnitine signature in liver corretales with non-alcoholic fatty liver disease progression. J Hepatol. (2022) 77:S681. doi: 10.1016/S0168-8278(22)01680-4

[27] Zvyagintseva TD Glushchenko SV. The effect of L-Carnitine and proinflammatory cytokines in the development of nonalcoholic steatohepatitis. Bangladesh J Med Sci. (2016) 15:62–5. doi: 10.3329/bjms.v15i1.20660

[28] Sun C Guo Y Cong P Tian Y Gao X. Liver lipidomics analysis revealed the novel ameliorative mechanisms of L-Carnitine on high-fat diet-induced NAFLD Mice. Nutrients. (2023) 15:1359. doi: 10.3390/nu15061359 36986087 PMC10053018

[29] Maeda H Hosomi R Fukuda M Ikeda Y Yoshida M Fukunaga K. Dietary Tuna Dark Muscle Protein Attenuates Hepatic Steatosis and Increases Serum High-Density Lipoprotein Cholesterol in Obese Type-2 Diabetic/Obese KK-A(y) Mice. J Food Sci. (2017) 82:1231–8. doi: 10.1111/1750-3841.13711 28422289

[30] Abd Eldaim MA Ibrahim FM Orabi SH Hassan A El Sabagh HS. l-Carnitine-induced amelioration of HFD-induced hepatic dysfunction is accompanied by a reduction in hepatic TNF-α and TGF-β1. Biochem Cell Biol. (2018) 96:713–25. doi: 10.1139/bcb-2018-0074 29677453

[31] Sunagozaka H Honda M Yamashita T Okada H Oishi N Shimakami T. et al. The L-carnitine alleviate hepatic fibrosis in a non-alcoholic steatohepatitis. Hepatology. (2015) 62:872A.

[32] Kathirvel E Morgan K French SW Morgan TR. Acetyl-L-carnitine and lipoic acid improve mitochondrial abnormalities and serum levels of liver enzymes in a mouse model of nonalcoholic fatty liver disease. Nutr Res. (2013) 33:932–41. doi: 10.1016/j.nutres.2013.08.001 24176233

[33] Hong JH Lee MK. Carnitine Orotate Complex Ameliorates Insulin Resistance and Hepatic Steatosis Through Carnitine Acetyltransferase Pathway. Diabetes Metab J. (2021) 45:933–47. doi: 10.4093/dmj.2020.0223 34407600 PMC8640142

[34] Kang JS Lee WK Yoon WK Kim N Park SK Park HK. et al. A combination of grape extract, green tea extract and L-carnitine improves high-fat diet-induced obesity, hyperlipidemia and non-alcoholic fatty liver disease in mice. Phytother Res. (2011) 25:1789–95. doi: 10.1002/ptr.3476 21480410

[35] Fujisawa K Takami T Matsuzaki A Matsumoto T Yamamoto N Terai S. et al. Evaluation of the effects of L-carnitine on medaka (Oryzias latipes) fatty liver. Sci Rep. (2017) 7:2749. doi: 10.1038/s41598-017-02924-5 28584294 PMC5459862

[36] Liu A Cai Y Yuan Y Liu M Zhang Z Xu Y. et al. Efficacy and safety of carnitine supplementation on NAFLD: a systematic review and meta-analysis. Syst Rev. (2023) 12:74. doi: 10.1186/s13643-023-02238-w 37120548 PMC10148537

[37] Malaguarnera M Gargante MP Russo C Antic T Vacante M Malaguarnera M. et al. L-carnitine supplementation to diet: a new tool in treatment of nonalcoholic steatohepatitis–a randomized and controlled clinical trial. Am J Gastroenterol. (2010) 105:1338–45. doi: 10.1038/ajg.2009.719 20068559

[38] Zakharova N Luo C Aringazina R Samusenkov V. The efficacy of L-carnitine in patients with nonalcoholic steatohepatitis and concomitant obesity. Lipids Health Dis. (2023) 22:101. doi: 10.1186/s12944-023-01867-3 37438785 PMC10337194

[39] Song JJ Ko KJ Cho YK Park HM Koo HM Kim NR. et al. Effectiveness of high-dose carnitine complex treatment in patients with nonalcoholic fatty liver disease: A retrospective, observational study. J Gastroenterol Hepatol. (2018) 33:423.

[40] Amiri-Moghadam S Nematy M Eghtesadi S Khalili M Mojarrad M Jazayeri S. et al. Effects of L-carnitine supplementation on body composition in patients with nonalcoholic steatohepatitis (NASH). Curr Top Nutraceutical Res. (2015) 13:71–6. doi: 10.1016/S0261-5614(17)30566-6

[41] Thiagarajan P Bawden S Simpson L Gowland P Greenhaff P Aithal GP. L-carnitine supplementation in non-alcoholic fatty liver disease: Effects on intrahepatic triglyceride, muscle lipid fractions and liver mitochondrial energetics-results from a pilot randomised trial. Hepatology. (2020) 72:1052A−3A. doi: 10.1136/gutjnl-2020-BASL.10

[42] Ashmore T Roberts LD Morash AJ Kotwica AO Finnerty J West JA. et al. Nitrate enhances skeletal muscle fatty acid oxidation via a nitric oxide-cGMP-PPAR-mediated mechanism. BMC Biol. (2015) 13:110. doi: 10.1186/s12915-015-0221-6 26694920 PMC4688964

[43] Montesano A Senesi P Vacante F Mollica G Benedini S Mariotti M. et al. L-Carnitine counteracts *in vitro* fructose-induced hepatic steatosis through targeting oxidative stress markers. J Endocrinol Invest. (2020) 43:493–503. doi: 10.1007/s40618-019-01134-2 31705397 PMC7067714

[44] Stefanovic-Racic M Perdomo G Mantell BS Sipula IJ Brown NF O'Doherty RM . moderate increase in carnitine palmitoyltransferase 1a activity is sufficient to substantially reduce hepatic triglyceride levels. Am J Physiol Endocrinol Metab. (2008) 294:E969–77. doi: 10.1152/ajpendo.00497.2007 18349115

[45] Ishikawa H Takaki A Tsuzaki R Yasunaka T Koike K Shimomura Y. et al. L-carnitine prevents progression of non-alcoholic steatohepatitis in a mouse model with upregulation of mitochondrial pathway. PLoS ONE. (2014) 9:e100627. doi: 10.1371/journal.pone.0100627 24983359 PMC4077577

[46] Ishikawa H Takaki A Yamamoto K. L-carnitine prevents progression of non-alcoholic steatohepatitis with regulation of mitochondrial β-oxidation and redox system in NASH model Mice. Hepatology. (2013) 58:561A.

[47] Mollica G Senesi P Codella R Vacante F Montesano A Luzi L. et al. L-carnitine supplementation attenuates NAFLD progression and cardiac dysfunction in a mouse model fed with methionine and choline-deficient diet. Dig Liver Dis. (2020) 52:314–23. doi: 10.1016/j.dld.2019.09.002 31607566

[48] Rodgers JT Lerin C Haas W Gygi SP Spiegelman BM Puigserver P. Nutrient control of glucose homeostasis through a complex of PGC-1alpha and SIRT1. Nature. (2005) 434:113–8. doi: 10.1038/nature03354 15744310

[49] Zhuge A Li S Han S Yuan Y Shen J Wu W. et al. Akkermansia muciniphila-derived acetate activates the hepatic AMPK/SIRT1/PGC-1α axis to alleviate ferroptosis in metabolic-associated fatty liver disease. Acta Pharm Sin B. (2025) 15:151–67. doi: 10.1016/j.apsb.2024.10.010 40041901 PMC11873632

[50] Purushotham A Schug TT Xu Q Surapureddi S Guo X Li X. Hepatocyte-specific deletion of SIRT1 alters fatty acid metabolism and results in hepatic steatosis and inflammation. Cell Metab. (2009) 9:327–38. doi: 10.1016/j.cmet.2009.02.006 19356714 PMC2668535

[51] Vega RB Huss JM Kelly DP. The coactivator PGC-1 cooperates with peroxisome proliferator-activated receptor alpha in transcriptional control of nuclear genes encoding mitochondrial fatty acid oxidation enzymes. Mol Cell Biol. (2000) 20:1868–76. doi: 10.1128/MCB.20.5.1868-1876.2000 10669761 PMC85369

[52] Song S Attia RR Connaughton S Niesen MI Ness GC Elam MB. et al. Peroxisome proliferator activated receptor alpha (PPARalpha) and PPAR gamma coactivator (PGC-1alpha) induce carnitine palmitoyltransferase IA (CPT-1A) via independent gene elements. Mol Cell Endocrinol. (2010) 325:54–63. doi: 10.1016/j.mce.2010.05.019 20638986 PMC3160239

[53] Hamza RT Elkabbany ZA Shedid AM Hamed AI Ebrahim AO. Serum chemerin in obese children and adolescents before and after L-Carnitine therapy: relation to nonalcoholic fatty liver disease and other features of metabolic syndrome. Arch Med Res. (2016) 47:541–9. doi: 10.1016/j.arcmed.2016.11.010 28262196

[54] Poulos JE Kalogerinis PT Milanov V Kalogerinis CT Poulos EJ. The Effects of Vitamin E, silymarin and carnitine on the metabolic abnormalities associated with nonalcoholic liver disease. J Diet Suppl. (2022) 19:287–302. doi: 10.1080/19390211.2021.1874587 33491528

[55] Poulos J Milanov V. Triple therapy utilizing vitamin E, milk thistle, and carnitine improves ALT and the metabolic abnormalities associated with NAFLD. Diabetes. (2016) 65:A617–A8.

[56] Bae J Lee W Yoon K Park J Son H Han K. et al. A multicentric, double-blind, randomised-controlled trial (RCT) of carnitine orotate complex in diabetic patients with non-alcoholic fatty liver disease (NAFLD). Diabetologia. (2014) 57:S349–S50.

[57] Bae JC Lee WY Yoon KH Park JY Son HS Han KA. et al. Improvement of Nonalcoholic Fatty Liver Disease With Carnitine-Orotate Complex in Type 2 Diabetes (CORONA): a randomized controlled trial. Diabetes Care. (2015) 38:1245–52. doi: 10.2337/dc14-2852 25877813

[58] Abolfathi M Mohd-Yusof BN Hanipah ZN Mohd Redzwan S Yusof LM Khosroshahi MZ. The effects of carnitine supplementation on clinical characteristics of patients with non-alcoholic fatty liver disease: a systematic review and meta-analysis of randomized controlled trials. Complement Ther Med. (2020) 48:102273. doi: 10.1016/j.ctim.2019.102273 31987257

[59] Amer AE Ghoneim HA Abdelaziz RR Shehatou GSG Suddek GM. L-carnitine attenuates autophagic flux, apoptosis, and necroptosis in rats with dexamethasone-induced non-alcoholic steatohepatitis. BMC Pharmacol Toxicol. (2024) 25:102. doi: 10.1186/s40360-024-00820-z 39736705 PMC11684100

[60] Sajan MP Standaert ML Nimal S Varanasi U Pastoor T Mastorides S. et al. The critical role of atypical protein kinase C in activating hepatic SREBP-1c and NFkappaB in obesity. J Lipid Res. (2009) 50:1133–45. doi: 10.1194/jlr.M800520-JLR200 19202134 PMC2681395

[61] Salic K Gart E Seidel F Verschuren L Caspers M van Duyvenvoorde W . Combined treatment with L-Carnitine and nicotinamide riboside improves hepatic metabolism and attenuates obesity and liver steatosis. Int J Mol Sci. (2019) 20: doi: 10.3390/ijms20184359 31491949 PMC6770226

[62] Terruzzi I Senesi P Montesano A Mazzilli M Luzi L. Effect of l-carnitine on hepatocyte insulin action and metabolism. Diabetes. (2015) 64:A181.

[63] Somi MH Fatahi E Panahi J Havasian MR Judaki A. Data from a randomized and controlled trial of L–Carnitine prescription for the treatment for Non- Alcoholic Fatty Liver Disease. Bioinformation. (2014) 10:575–9. doi: 10.6026/97320630010575 25352725 PMC4209366

[64] Hazzan R Abu Ahmad N Slim W Mazen E Neeman Z. Hepatoprotective effect of combination of L-carnitine and magnesium-hydroxide in nonalcoholic fatty liver disease patients: a double-blinded randomized controlled pilot study. Eur Rev Med Pharmacol Sci. (2022) 26:7522–32. doi: 10.26355/eurrev_202210_30023 36314323

[65] Mohammadi F Ghalavand A Delaramnasab M. Effect of circuit resistance training and l-carnitine supplementation on body composition and liver function in men with non-alcoholic fatty liver disease. Jundishapur J Chronic Dis Care. (2019) 8: doi: 10.5812/jjcdc.90213

[66] Alavinejad P Zakerkish M Eskandar H. Hashemi sJ, Chobineh M, Moghaddam E. Evaluation of L-Carnitine efficacy in the treatment of non- alcoholic fatty liver disease among diabetic patients: a randomized double blind pilot study. J Gastroenterol Hepatol Res. (2016) 5:2191–5. doi: 10.17554/j.issn.2224-3992.2016.05.662

[67] Hong ES Kim EK Kang SM Khang AR Choi SH Park KS. et al. Effect of carnitine-orotate complex on glucose metabolism and fatty liver: a double-blind, placebo-controlled study. J Gastroenterol Hepatol. (2014) 29:1449–57. doi: 10.1111/jgh.12536 24611967

[68] Saneian H Khalilian L Heidari-Beni M Khademian M Famouri F Nasri P. et al. Effect of l-carnitine supplementation on children and adolescents with nonalcoholic fatty liver disease (NAFLD): a randomized, triple-blind, placebo-controlled clinical trial. J Pediatr Endocrinol Metab. (2021) 34:897–904. doi: 10.1515/jpem-2020-0642 33939897

[69] Mu C Wang S Wang Z Tan J Yin H Wang Y. et al. Mechanisms and therapeutic targets of mitochondria in the progression of metabolic dysfunction-associated steatotic liver disease. Ann Hepatol. (2025) 30:101774. doi: 10.1016/j.aohep.2024.101774 39701281

[70] Meng D Chang M Dai X Kuang Q Wang G. GTPBP8 mitigates nonalcoholic steatohepatitis (NASH) by depressing hepatic oxidative stress and mitochondrial dysfunction via PGC-1α signaling. Free Radic Biol Med. (2025) 229:312–32. doi: 10.1016/j.freeradbiomed.2024.09.044 39341301

[71] Zhang M Ji J Lei Y Qin F Tao Y Li N. et al. Dual inhibition of hepatic ACLY and ACSS2: A synergistic approach to combat NAFLD through lipogenesis reduction and mitochondrial enhancement. Pharmacol Res. (2025) 215:107706. doi: 10.1016/j.phrs.2025.107706 40127788

[72] Jun DW Cho WK Jun JH Kwon HJ Jang KS Kim HJ. et al. Prevention of free fatty acid-induced hepatic lipotoxicity by carnitine via reversal of mitochondrial dysfunction. Liver Int. (2011) 31:1315–24. doi: 10.1111/j.1478-3231.2011.02602.x 22093454

[73] Lyu J Okada H Sunagozaka H Kawaguchi K Shimakami T Nio K . Potential utility of l-carnitine for preventing liver tumors derived from metabolic dysfunction-associated steatohepatitis. Hepatol Commun. (2024) 8:e0425. doi: 10.1097/HC9.0000000000000425 38619434 PMC11019826

[74] Lim CY Jun DW Jang SS Cho WK Chae JD Jun JH. Effects of carnitine on peripheral blood mitochondrial DNA copy number and liver function in non-alcoholic fatty liver disease. Korean J Gastroenterol. (2010) 55:384–9. doi: 10.4166/kjg.2010.55.6.384 20571306

[75] Chang B Nishikawa M Nishiguchi S Inoue M. L-carnitine inhibits hepatocarcinogenesis via protection of mitochondria. Int J Cancer. (2005) 113:719–29. doi: 10.1002/ijc.20636 15499623

[76] Pradhany RC Siswanto FM Sukoco H Nyoman Suarsana I Gusti Ayu Agung Suartini I. L-carnitine Prevents Hepatic Steatosis in Deep-Frying Oil-Treated Rat. Biomed Pharmacol J. (2022) 15:1751–8. doi: 10.13005/bpj/2514

[77] Zeng X Sheng X. The restorative effect of l-carnitine on experimental nonalcoholic steatohepatitis. Gastroenterology. (2013) 144:S1022. doi: 10.1016/S0016-5085(13)63795-6

[78] Yu Y Liu Y An W Song J Zhang Y Zhao X. STING-mediated inflammation in Kupffer cells contributes to progression of nonalcoholic steatohepatitis. J Clin Invest. (2019) 129:546–55. doi: 10.1172/JCI121842 30561388 PMC6355218

[79] Kang JS Lee WK Lee CW Yoon WK Kim N Park SK. et al. Improvement of high-fat diet-induced obesity by a mixture of red grape extract, soy isoflavone and L-carnitine: implications in cardiovascular and non-alcoholic fatty liver diseases. Food Chem Toxicol. (2011) 49:2453–8. doi: 10.1016/j.fct.2011.06.071 21745528

[80] Nofal AE AboShabaan HS Fadda WA Ereba RE Elsharkawy SM Hathout HM. L-carnitine and Ginkgo biloba Supplementation *In Vivo* Ameliorates HCD-Induced Steatohepatitis and Dyslipidemia by Regulating Hepatic Metabolism. Cells. (2024) 13:732. doi: 10.3390/cells13090732 38727268 PMC11083725

[81] Feng X Weng D Zhou F Owen YD Qin H Zhao J. et al. Activation of PPARγ by a Natural Flavonoid Modulator, Apigenin Ameliorates Obesity-Related Inflammation Via Regulation of Macrophage Polarization. EBioMedicine. (2016) 9:61–76. doi: 10.1016/j.ebiom.2016.06.017 27374313 PMC4972579

[82] Eghtesadi S Amiri Moghadam S Nemati M Khalili M Mojarrad M Jazayeri S. et al. Effects of L-carnitine supplementation on inflammatory factors and malondialdehyde in patients with nonalcoholic steatohepatitis (NASH). Obesity Facts. (2016) 9:184.

[83] Terayama Y Nakamura SI Mekada K Matsuura T Ozaki K. High-fat diet-induced nonalcoholic steatohepatitis is accelerated by low carnitine and impaired glucose tolerance in novel murine models. Lab Invest. (2022) 102:621–30. doi: 10.1038/s41374-022-00732-8 35039610

[84] Matter MS Marquardt JU Andersen JB Quintavalle C Korokhov N Stauffer JK. et al. Oncogenic driver genes and the inflammatory microenvironment dictate liver tumor phenotype. Hepatology. (2016) 63:1888–99. doi: 10.1002/hep.28487 26844528 PMC4874846

[85] Huang H Liu N Guo H Liao S Li X Yang C. et al. L-carnitine is an endogenous HDAC inhibitor selectively inhibiting cancer cell growth *in vivo* and *in vitro*. PLoS ONE. (2012) 7:e49062. doi: 10.1371/journal.pone.0049062 23139833 PMC3489732

[86] Rinella ME Neuschwander-Tetri BA Siddiqui MS Abdelmalek MF Caldwell S Barb D. et al. AASLD Practice Guidance on the clinical assessment and management of nonalcoholic fatty liver disease. Hepatology. (2023) 77:1797–835. doi: 10.1097/HEP.0000000000000323 36727674 PMC10735173

[87] EASL-EASD-EASO. Clinical practice guidelines on the management of metabolic dysfunction-associated steatotic liver disease (MASLD). Obes Facts. (2024) 17:374-444. doi: 10.1159/000539371 PMC11299976 38852583

[88] Petta S Targher G Romeo S Pajvani UB Zheng MH Aghemo A. et al. The first MASH drug therapy on the horizon: Current perspectives of resmetirom. Liver Int. (2024) 44:1526–36. doi: 10.1111/liv.15930 38578141

[89] Li JM Li LY Zhang YX Jiang ZY Limbu SM Qiao F. et al. Functional differences between l- and d-carnitine in metabolic regulation evaluated using a low-carnitine Nile tilapia model. Br J Nutr. (2019) 122:625–38. doi: 10.1017/S000711451900148X 32124711

[90] Su X Chen S Liu J Feng Y Han E Hao X. et al. Composition of gut microbiota and non-alcoholic fatty liver disease: a systematic review and meta-analysis. Obes Rev. (2024) 25:e13646. doi: 10.1111/obr.13646 37813400

[91] Zhang D Leitman M Pawar S Shera S Hernandez L Jacobs JP . The association between prevotella copri and advanced fibrosis in the progression of metabolic dysfunction-associated steatotic liver disease. Nutrients. (2025) 17:2145. doi: 10.3390/nu17132145 40647253 PMC12251637

[92] Quesada-Vázquez S Bone C Saha S Triguero I Colom-Pellicer M Aragonès G . Microbiota dysbiosis and gut barrier dysfunction associated with non-alcoholic fatty liver disease are modulated by a specific metabolic cofactors' combination. Int J Mol Sci. (2022) 23:13675. doi: 10.3390/ijms232213675 36430154 PMC9692973

[93] Pierantonelli I Rychlicki C Agostinelli L Giordano DM Gaggini M Fraumene C. et al. Lack of NLRP3-inflammasome leads to gut-liver axis derangement, gut dysbiosis and a worsened phenotype in a mouse model of NAFLD. Sci Rep. (2017) 7:12200. doi: 10.1038/s41598-017-11744-6 28939830 PMC5610266

[94] Ishikawa H. Vitamin e and l-carnitine prevents progression of non-alcoholic steatohepatitis with regulation of intestinal inflammasome activation in nash model mice. Gastroenterology. (2013) 144:S1019. doi: 10.1016/S0016-5085(13)63782-8

[95] Xie Z Li H Wang K Lin J Wang Q Zhao G. et al. Analysis of transcriptome and metabolome profiles alterations in fatty liver induced by high-fat diet in rat. Metabolism. (2010) 59:554–60. doi: 10.1016/j.metabol.2009.08.022 19913842

[96] Socha P Horvath A Vajro P Dziechciarz P Dhawan A Szajewska H. Pharmacological interventions for nonalcoholic fatty liver disease in adults and in children: a systematic review. J Pediatr Gastroenterol Nutr. (2009) 48:587–96. doi: 10.1097/MPG.0b013e31818e04d1 19412008

[97] Li N Zhao H. Role of carnitine in non-alcoholic fatty liver disease and other related diseases: an update. Front Med. (2021) 8:689042. doi: 10.3389/fmed.2021.689042 34434943 PMC8381051

[98] Savic D Hodson L Neubauer S Pavlides M. The Importance of the Fatty Acid Transporter L-Carnitine in Non-Alcoholic Fatty Liver Disease (NAFLD). Nutrients. (2020) 12:2178. doi: 10.3390/nu12082178 32708036 PMC7469009

